# Genome-Based Taxonomy of *Brevundimonas* with Reporting *Brevundimonas huaxiensis* sp. nov

**DOI:** 10.1128/spectrum.00111-21

**Published:** 2021-07-07

**Authors:** Lina Liu, Yu Feng, Li Wei, Zhiyong Zong

**Affiliations:** a Center of Infectious Diseases, West China Hospital, Sichuan University, Chengdu, China; b Center for Pathogen Research, West China Hospital, Sichuan University, Chengdu, China; c Division of Infectious Diseases, State Key Laboratory of Biotherapy, Chengdu, China; d Department of Infection Control, West China Hospital, Sichuan University, Chengdu, China; The Pennsylvania State University

**Keywords:** *Brevundimonas huaxiensis*, taxonomy, species assignation, genome-based taxonomy, genomics

## Abstract

*Brevundimonas* is a genus of Gram-negative bacteria widely distributed in nature and is also an opportunistic pathogen causing health care-associated infections. *Brevundimonas* strain 090558^T^ was recovered from a blood culture of a cancer patient and was subjected to genome sequencing and analysis. The average nucleotide identity and *in silico* DNA-DNA hybridization values between 090558^T^ and type strains of *Brevundimonas* species were 78.76% to 93.94% and 19.8% to 53.9%, respectively, below the cutoff to define bacterial species. Detailed phenotypic tests were performed, suggesting that 090558^T^ can be differentiated from other *Brevundimonas* species by its ability to assimilate sodium acetate but not to utilize glucose, trypsin, or β-glucosidase. Strain 090558^T^ (GDMCC 1.1871^T^ or KCTC 82165^T^) therefore represents a novel *Brevundimonas* species, for which the name Brevundimonas huaxiensis sp. nov. is proposed. All *Brevundimonas* genomes available in GenBank (accessed on 25 January 2021) were retrieved, discarding those labeled “excluded from RefSeq” by GenBank, and included 82 genomes for precise species curation. In addition to the 21 *Brevundimonas* species with genomes of type strains available, we identified 29 *Brevundimonas* taxa that either belong to the 12 *Brevundimonas* species without available genomes of type strains or represent novel species. We found that more than half (57.3%) of the 82 *Brevundimonas* genomes need to be corrected for species assignation, including species mislabeling of a type strain. Our analysis highlights the complexity of *Brevundimonas* taxonomy. We also found that only some *Brevundimonas* species are associated with human infections, and more studies are warranted to understand their pathogenicity and epidemiology.

**IMPORTANCE** *Brevundimonas* is a genus of the family *Caulobacteraceae* and comprises 33 species. *Brevundimonas* can cause various infections but remains poorly studied. In this study, we reported a novel *Brevundimonas* species, *Brevundimonas huaxiensis*, based on genome and phenotype studies of strain 090558^T^ recovered from human blood. We then examined the species assignations of all *Brevundimonas* genomes (*n* = 82) in GenBank and found that in addition to the known *Brevundimonas* species with genome sequences of type strains available, there are 29 *Brevundimonas* taxa based on genome analysis, which need to be further studied using phenotype-based methods to establish their species status. Our study significantly updates the taxonomy of *Brevundimonas* and enhances our understanding of this genus of clinical relevance. The findings also encourage future studies on the characterization of novel *Brevundimonas* species.

## INTRODUCTION

The genus *Brevundimonas* was first proposed by Segers et al. (1). *Brevundimonas* strains are Gram-negative, motile bacteria of the family *Caulobacteraceae*; contain Q-10 as the major isoprenoid quinone; and have high DNA G+C contents (2–4). At the time of writing, the genus *Brevundimonas* comprises 33 species (Table 1). *Brevundimonas* can be found in diverse environments such as soils (5), water (3, 6, 7), activated sludge (8), and plant roots (9). *Brevundimonas* has the potential to be used for a wide range of activities such as cadmium biosorption (10), soil bioremediation (11), water pollutant treatment (12), and plant growth promotion for sustainable agriculture in arid regions (13). In humans, *Brevundimonas* is an opportunistic pathogen able to cause a range of hospital-acquired infections such as bacteremia, eye infection, peritonitis, urinary tract infection, and skin and soft tissue infection (14). In this study, we report a clinical strain from blood to represent a novel *Brevundimonas* species, Brevundimonas huaxiensis, based on genome and phenotype studies. We also curated all *Brevundimonas* genomes available in GenBank for precise species identification and found at least 17 tentative novel *Brevundimonas* species.

**TABLE 1:** Nucleotide identities of 16S rRNA gene sequences between strain 090558^T^ and type strains of *Brevundimonas* species^*a*^

Species	Strain	GenBank accession no.	Identity (%)
***B. vesicularis***	**NBRC 12165^T^**	**AB680247**	**99.71**
***B. nasdae***	**JCM 11415^T^**	**AB071954**	**99.64**
***B. intermedia***	**ATCC 15262^T^**	**AJ227786**	**99.49**
***B. aurantiaca***	**DSM 4731^T^**	**AJ227787**	**99.13**
***B. mediterranea***	**V4.BO.10^T^**	**AJ227801**	**98.91**
*B. olei*	MJ15^T^	GQ250440	98.34
*B. albigilva*	NHI-13^T^	KC733808	98.27
*B. naejangsanensis*	DSM 23858^T^	ATXN01000003	97.98
*B. kwangchunensis*	KSL-102^T^	AY971368	97.76
*B. viscosa*	CGMCC 1.10683^T^	jgi.1076140	97.69
*B. humi*	CA-15^T^	KY117472	97.61
*B. faecalis*	CS20.3^T^	FR775448	97.58
*B. alba*	DSM 4736^T^	AJ227785	97.40
*B. bacteroides*	DSM 4726^T^	JNIX01000007	97.39
*B. poindexterae*	FWC40^T^	AJ227797	97.18
*B. halotolerans*	MCS24^T^	QTTA01000016	97.18
*B. diminuta*	ATCC 11568^T^	GL883089	97.18
*B. bullata*	IAM 13153^T^	D12785	97.18
*B. vancanneytii*	LMG 2337^T^	AJ227779	97.11
*B. balnearis*	FDRGB2b^T^	LN651199	97.11
*B. staleyi*	FWC43^T^	AJ227798	97.11
*B. lenta*	DS-18^T^	EF363713	96.97
*B. terrae*	KSL-145^T^	DQ335215	96.89
*B. denitrificans*	TAR-002^T^	AB899817	96.89
*B. subvibrioides*	ATCC 15264^T^	ADBM01000034	96.89
*B. fluminis*	LA-55^T^	RQWJ01000003	96.82
*B. variabilis*	ATCC 15255^T^	AJ227783	96.75
*B. basaltis*	J22^T^	EU143355	96.64
*B. mongoliensis*	R-10-10^T^	MF436701	96.61
*B. aveniformis*	DSM 17977^T^	AUAO01000001	96.58
*B. lutea*	NS26^T^	KX601076	95.12
*B. abyssalis*	TAR-001^T^	BATC01000012	94.88
*B. canariensis*	GTAE24^T^	KX898252	94.73

a Type strains having >98.5% 16S rRNA gene sequence identity with strain 090558^T^ are highlighted in boldface type.

## RESULTS

### Strain 090558^T^ represents a novel *Brevundimonas* species with the proposed name *Brevundimonas huaxiensis*.

Strain 090558^T^ was recovered from a blood culture of a 46-year-old male patient with hepatocellular carcinoma. The patient received liver surgery 18 days prior to the collection of the blood culture but had no central lines, drainage tubes, or any other invasive devices within 2 weeks before the blood culture. The source of this strain is unknown. The strain was preliminarily identified as Brevundimonas vesicularis by Vitek II (bioMérieux, Marcy l’Etoile, France). The strain was susceptible to amikacin, ampicillin, ampicillin-sulbactam, aztreonam, ceftriaxone, ceftazidime, cefepime, cefotaxime, cefuroxime, chloramphenicol, ciprofloxacin, colistin, imipenem, meropenem, piperacillin-tazobactam, sulfamethoxazole-trimethoprim, and tigecycline but resistant to aztreonam. The patient responded well to treatment with cefoperazone-sulbactam and was discharged from the hospital later.

**FIG 1:**
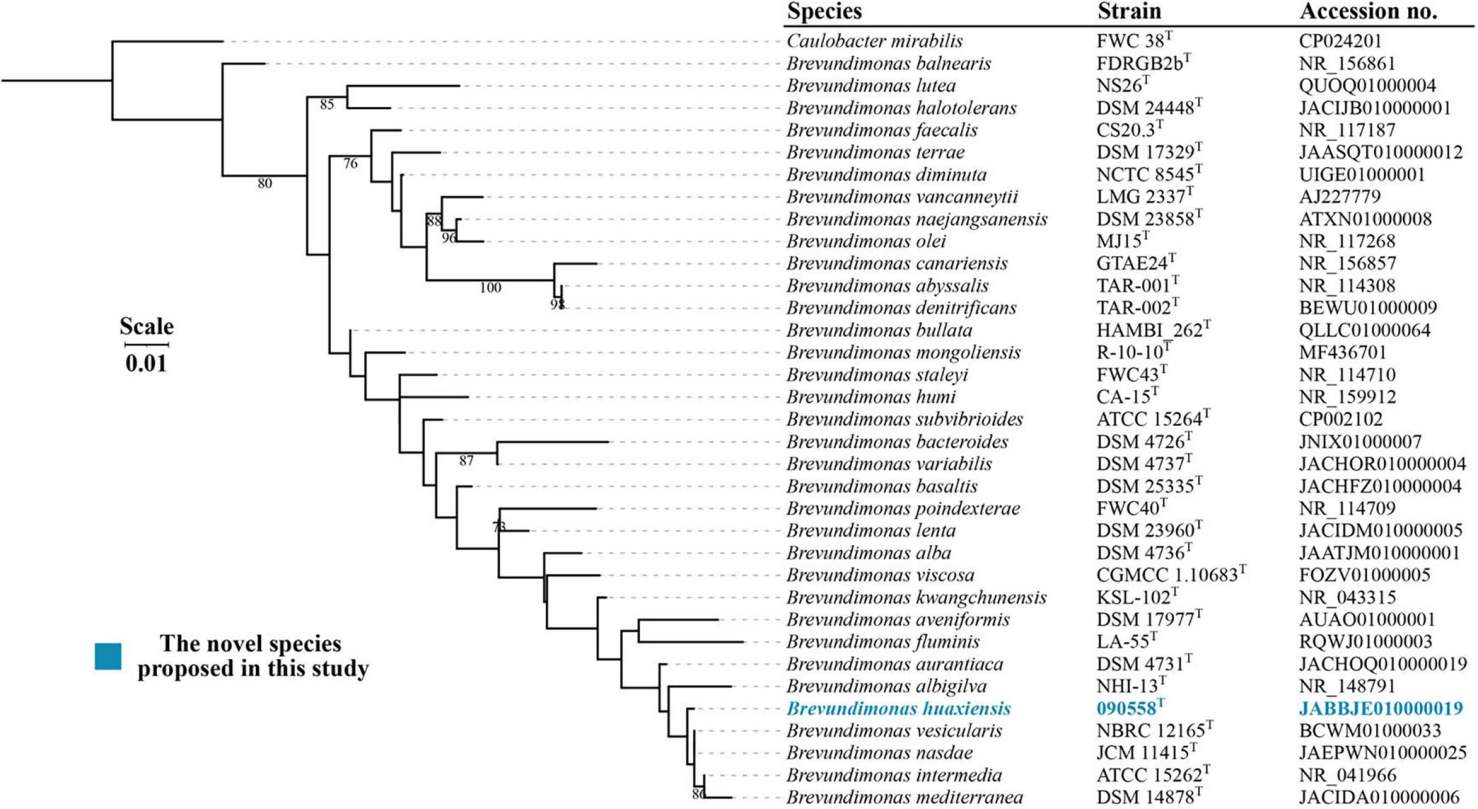
Phylogenetic tree based on 16S rRNA gene sequences of strain 090558^T^ and type strains of *Brevundimonas* species (Table 1). The tree was inferred using the maximum likelihood method. Caulobacter mirabilis FWC 38^T^ (GenBank accession no. CP024201) was used as an outgroup. Bootstrap values of >70% (based on 1,000 resamplings) are shown. Bar, 0.01 substitutions per nucleotide position.

The nearly complete 16S rRNA gene sequence of strain 090558^T^ (1,383 bp) was obtained, which had the highest identity to those of Brevundimonas vesicularis NBRC 12165^T^ (99.71%), Brevundimonas nasdae JCM 11415^T^ (99.64%), Brevundimonas intermedia ATCC 15262^T^ (99.49%), Brevundimonas aurantiaca DSM 4731^T^ (99.13%), and Brevundimonas mediterranea V4.BO.10^T^ (98.91%) (Table 1). However, strain 090558^T^ appears to be well separated from other *Brevundimonas* species in the phylogenetic tree based on the 16S rRNA gene sequence (see Fig. 1 for the maximum likelihood tree; the neighbor-joining and maximum parsimony trees are shown in Fig. S1 and S2 in the supplemental material). It is well known that analysis based on 16S rRNA gene sequences is not sufficient for precise species identification of bacterial species (15). We therefore performed whole-genome sequencing using a short-read Illumina sequencer for strain 090558^T^.

A total of 6,478,361 reads and 1.94 GB of clean bases were generated, which were then assembled into 35 contigs (*N*_50_, 355,886 bp). The draft genome of strain 090558^T^ is 3,163,842 bp in size with a 66.4 mol% G+C content. JCM 11415^T^, the type strain of B. nasdae, had >99% 16S rRNA sequence identity with strain 090558^T^ (see below) but had no genome sequences or sequences of housekeeping genes for comparison. We therefore obtained *B. nasdae* type strain JCM 11415^T^ and also sequenced the strain using HiSeq X10, which generated a total of 5,993,515 reads and 1.80 GB of clean bases, which were then assembled into 39 contigs (*N*_50_, 260,309 bp). The draft genome of strain JCM 11415^T^ is 3,751,817 bp in size with a 65.6 mol% G+C content.

**FIG 2:**
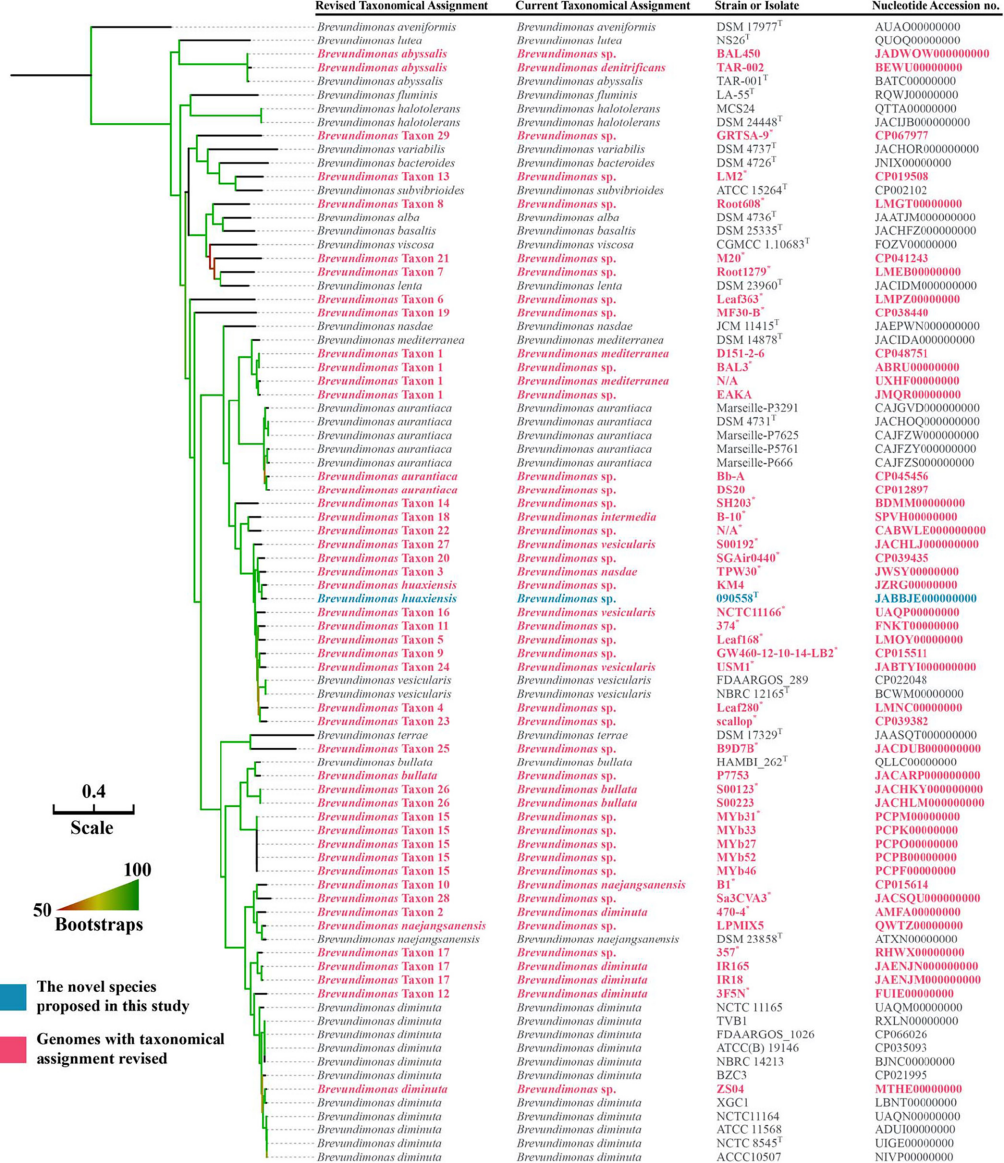
Phylogenomic tree based on the concatenated nucleotide sequences of core genes of strain 090558^T^ and all *Brevundimonas* genomes available in GenBank (accessed on 25 January 2021) (see Data Set S1 in the supplemental material). Strains and their nucleotide accession numbers are listed alongside the labeled species names in GenBank and precise species names assigned in this study. The tree was inferred using the maximum likelihood method under a GTR gamma model with a 1,000-bootstrap test, and branches with support of over 50% are indicated by gradients. The bar indicates nucleotide substitutions per site.

In the maximum likelihood phylogenomic tree (Fig. 2) based on the 1,040 core genes of all *Brevundimonas* genomes available in GenBank (see Data Set S1 in the supplemental material), strain 090558^T^ was well situated within the genus *Brevundimonas* and was most closely related to B. vesicularis among known *Brevundimonas* species. The average nucleotide identity (ANI) values between strain 090558^T^ and the type strains of all *Brevundimonas* species with genome sequences ranged from 78.76% to 93.94%, lower than the ≥95 to 96% cutoff for defining species (16) (Table 2). Consistently, the *in silico* DNA-DNA hybridization (isDDH) values between strain 090558^T^ and the type strains of all *Brevundimonas* species with genome sequences ranged from 19.8% to 53.9% (Table 2), which are below the ≥70.0% cutoff to define species (16, 17). The optimal isDDH cutoff to define a bacterial genus has not been established, and relatively low (around 20%) isDDH values between species within the same genus have been reported previously, such as for Acinetobacter (18) and Pseudomonas (19). Of note, among the five species having >98.5% 16S rRNA gene sequence identity with strain 090558^T^, the type strain of B. intermedia has no available genome sequences. Nonetheless, the housekeeping gene *gyrB* (encoding DNA gyrase subunit B) of strain 090558^T^ had only 82.19% nucleotide identity to that of the *B. intermedia* CIP 106444^T^ (GenBank accession no. EU024185). This suggests that strain 090558^T^ does not belong to *B. intermedia*.

**TABLE 2:** ANI and isDDH values between 090558^T^ and type strains of *Brevundimonas* species^*a*^

Species	Strain	GenBank accession no.	ANI (%)	isDDH value (%)
*B. abyssalis*	TAR-001^T^	BATC00000000	80.28	20.30
*B. alba*	DSM 4736^T^	JAATJM000000000	81.08	21.00
***B. aurantiaca***	**DSM 4731^T^**	**JACHOQ000000000**	**85.40**	**26.40**
*B. aveniformis*	DSM 17977^T^	AUAO00000000	78.76	20.80
*B. bacteroides*	DSM 4726^T^	JNIX00000000	80.70	20.60
*B. basaltis*	DSM 25335^T^	JACHFZ000000000	80.92	20.90
*B. bullata*	HAMBI 262^T^	QLLC00000000	82.65	21.80
*B. diminuta*	ATCC 11568^T^	GL883089	82.46	22.10
*B. fluminis*	LA-55^T^	RQWJ00000000	80.58	20.50
*B. halotolerans*	MCS24^T^	QTTA01000000	80.47	20.20
*B. lenta*	DSM 23960^T^	JACIDM000000000	81.74	21.70
*B. lutea*	NS26^T^	QUOQ00000000	80.21	20.80
***B. mediterranea***	**DSM 14878^T^**	**JACIDA000000000**	**86.16**	**27.60**
*B. naejangsanensis*	DSM 23858^T^	ATXN00000000	82.13	21.30
***B. nasdae***	**JCM 11415^T^**	**JAEPWN000000000**	**85.25**	**25.80**
*B. subvibrioides*	ATCC 15264^T^	ADBM00000000	81.19	21.00
*B. terrae*	DSM 17329^T^	JAASQT000000000	78.98	19.80
*B. variabilis*	DSM 4737^T^	JACHOR000000000	79.84	20.10
***B. vesicularis***	**NBRC 12165^T^**	**BCWM00000000**	**93.94**	**53.90**
*B. viscosa*	CGMCC 1.10683^T^	FOZV00000000	80.83	20.70

a Type strains having >98.5% 16S rRNA gene sequence identity with strain 090558^T^ (Table 1) are highlighted in boldface type.

The above-described analyses clearly suggest that strain 090558^T^ represents a novel species of the genus *Brevundimonas*. As the strain was recovered from West China Hospital, we propose the species name *Brevundimonas huaxiensis* (hua.xi.en′sis. fem. adj. *huaxiensis*, referring to West China [*huaxi* in Chinese], where the type strain was recovered) after phenotypic characterization (see below). Strain 090558^T^ has been deposited into the Guangdong Microbial Culture Collection Center as GDMCC1.1871^T^ and into the Korean Collection for Type Cultures as KCTC 82165^T^.

### *B. huaxiensis* can be differentiated from other *Brevundimonas* species by its ability to assimilate sodium acetate but not to utilize glucose, trypsin, or β-glucosidase.

Cells of strain 090558^T^ were Gram stain negative, aerobic, motile, non-spore forming, and rod shaped (0.3 to 0.5 μm in diameter and 1.0 to 2.5 μm long) (Fig. S3). Strain 090558^T^ grew on nutrient agar, tryptic soy agar, R2A agar, MacConkey agar, and brain heart infusion (BHI) agar after 2 days. Colonies grown on nutrient agar at 35°C after 2 days were circular, smooth, convex, and orange. Growth occurs at 8°C to 42°C in the presence of 0 to 4% (wt/vol) NaCl and at pH 6.0 to 8.0. It is negative for oxidase and activities of arylamidase, arginine dihydrolase, cystine, α-chymotrypsin, α-fucosidase, α-galactosidase, β-galactosidase, β-glucuronidase, β-glucosidase, lipase (C_14_), α-mannosidase, *N*-acetyl-β-glucosaminidase, and trypsin. Catalase, PNPG (*p*-nitrophenyl-β-d-galactopyranoside), indole production, and nitrate reduction tests and activities of acid phosphatase, alkaline phosphatase, esterase (C_4_), esterase lipase (C_8_), α-glucosidase, leucine arylamidase, naphthol-AS-BI(7-bromo-3-hydroxy-2-naphtho-o-anisidine)-phosphohydrolase, urease, and valine arylamidase are positive. Tween 40, esculin, and gelatin are hydrolyzed, but starch, DNase, and cellulose are not. It is able to assimilate *N*-acetylglucosamine, potassium gluconate, and sodium acetate but does not utilize adipic acid, arabinose, capric acid, glucose, malic acid, maltose, mannose, mannitol, phenylacetic acid, and trisodium citrate. It can assimilate sodium acetate instead of glucose. It is negative for oxidase and activities of trypsin and β-glucosidase. The ability to assimilate sodium acetate but not to utilize glucose, trypsin, or β-glucosidase can differentiate 090558^T^ from other *Brevundimonas* species (Table 3).

**TABLE 3:** Phenotypic characteristics of strain 090558^T^ and closely related strains of the genus *Brevundimonas*^*a*^

Characteristic	Result or value for strain					
	090558^T^	*B. aurantiaca*	*B. vesicularis*	*B. intermedia*	*B. nasdae*	*B. mediterranea*
Colony color	Orange	Yellow	Orange	Cream	Slightly yellow	Cream white

Property						
Motility	+	+	+	+	+	+
Esculin hydrolysis	+	+	+	+	+	−
PNPG	+	−	+	−	+	−
Assimilation of:						
Glucose	−	+	+	−	+	−
Mannose	−	−	−	−	−	−
*N*-Acetylglucosamine	+	−	W	−	+	−
Maltose	−	W	+	−	+	−
Malic acid	−	W	+	−	+	+
Trisodium citrate	−	−	−	−	−	−
Phenylacetic acid	−	−	−	−	−	−
Sodium acetate	+	−	−	−	+	+
Enzyme activity						
Oxidase	−	+	+	+	+	+
Catalase	+	+	+	+	+	−
Lipase (C_14_)	−	−	W	−	W	−
Leucine arylamidase	+	+	+	W	W	+
Valine arylamidase	+	W	W	+	+	W
Trypsin	−	+	W	W	+	+
α-Chymotrypsin	−	W	W	W	W	W
Acid phosphatase	+	+	+	+	+	+
α-Galactosidase	−	−	−	−	+	−
β-Galactosidase	−	W	+	W	W	−
β-Glucuronidase	−	−	−	−	−	−
α-Glucosidase	+	W	W	W	+	−
β-Glucosidase	−	−	+	−	+	−

G+C content (mol%)	66.4	65.6	65.0–66.0	66.1	66.5	67.3

a +, positive; −, negative; W, weakly positive. Data for species other than *B. huaxiensis* are from reference 2. Closely related species refer to those having >98.5% 16S rRNA gene sequence identity with strain 090558^T^ (Table 1).

The isoprenoid quinone of strain 090558^T^ was Q-10, which is typical of members of the genus *Brevundimonas*. The major cellular fatty acids were summed feature 8 (comprising C_18:1_ ω7c/C_18:1_ ω6c) (55.87%) and C_16:0_ (25.2%), which are the same as those of closely related *Brevundimonas* species (Table S1).

### Curation of *Brevundimonas* genomes available in GenBank.

With the inclusion of *B. huaxiensis* identified in this study, there are 34 known *Brevundimonas* species at present (Table 4). Genome sequences of type strains are available for 22 *Brevundimonas* species, including *B. huaxiensis* 090558^T^ and *B. nasdae* JCM 11415^T^ sequenced in this study. We then determined pairwise ANI and isDDH values between the type strain genomes. We found that the genome of Brevundimonas denitrificans strain TAR-002^T^ (GenBank accession no. BEWU00000000) had 99.78% ANI and 99.1% isDDH with that of Brevundimonas abyssalis TAR-001^T^ (GenBank accession no. BATC00000000) (Data Set S2), suggesting that the two strains actually belonged to the same species or that the strains were mislabeled. We therefore checked the identity of the two genomes with their corresponding 16S rRNA gene sequences available in GenBank and found only 93.97% identity between the genome of TAR-002^T^ and its original 16S rRNA sequence (GenBank accession no. AB899817). It is therefore likely that the genome of TAR-002^T^ was mislabeled for another strain of *B. abyssalis*. Genome sequences of type strains of the remaining 20 species have <95% ANI and <70% isDDH values between each other (Data Set S2).

**TABLE 4:** Updated taxonomy of *Brevundimonas*, including 34 species at present

Species	Strain	GenBank accession no. of genome sequence	Reference
*B. abyssalis*	TAR-001^T^	BATC00000000	39
*B. alba*	DSM 4736^T^	JAATJM000000000	40
*B. albigilva*	NHI-13^T^		2
*B. aurantiaca*	DSM 4731^T^	JACHOQ000000000	40
*B. aveniformis*	DSM 17977^T^	AUAO00000000	8
*B. bacteroides*	DSM 4726^T^	AUAO00000000	40
*B. balnearis*	FDRGB2b^T^		3
*B. basaltis*	J22^T^	JACHFZ000000000	41
*B. bullata*	IAM 13153^T^	QLLC00000000	42
*B. canariensis*	GTAE24^T^		9
*B. denitrificans*	TAR-002^T^	—^*a*^	43
*B. diminuta*	ATCC 11568^T^	GL883089	1
*B. faecalis*	CS20.3^T^		44
*B. fluminis*	LA-55^T^	RQWJ00000000	6
*B. halotolerans*	MCS24^T^	QTTA00000000	45
*B. huaxiensis*	090558^T^	JABBJE000000000	This study
*B. humi*	CA-15^T^		5
*B. intermedia*	ATCC 15262^T^		40
*B. kwangchunensis*	KSL-102^T^		46
*B. lenta*	DS-18^T^	JACIDM000000000	47
*B. lutea*	NS26^T^	QUOQ00000000	7
*B. mediterranea*	V4.BO.10^T^	JACIDA000000000	4
*B. mongoliensis*	R-10-10^T^		48
*B. naejangsanensis*	DSM 23858^T^	ATXN00000000	42
*B. nasdae*	JCM 11415^T^	JAEPWN000000000	49
*B. olei*	MJ15^T^		50
*B. poindexterae*	FWC40^T^		45
*B. staleyi*	FWC43^T^		45
*B. subvibrioides*	ATCC 15264^T^	CP002102	40
*B. terrae*	KSL-145^T^	JAASQT000000000	51
*B. vancanneytii*	LMG 2337^T^		25
*B. variabilis*	ATCC 15255^T^	JACHOR000000000	40
*B. vesicularis*	NBRC 12165^T^	BCWM00000000	1
*B. viscosa*	CGMCC 1.10683^T^	FOZV00000000	52

a GenBank accession no. BEWU00000000 of TAR-002^T^ is mislabeled and actually belongs to *B. abyssalis*.

There were 60 genome sequences of *Brevundimonas* non-type strains that were also available in GenBank. Among the 60 genomes, based on ANI and isDDH analyses with type strains of *Brevundimonas* species, 24 could be assigned to a known *Brevundimonas* species, while 36 could not (Data Set S1). Instead, a total of 29 taxa, which do not belong to any of the 21 *Brevundimonas* species with genome sequences of type strains available, could be identified from the 36 genomes and are assigned as taxa 1 to 29 here (Data Set S1). The closest species of the 29 taxa are listed in Table 5. More than half (*n* = 47 [57.3%; 47/82]) of the 82 available *Brevundimonas* genomes need to be corrected for species assignation. It is suggested based on the genome comparison reported here that (i) 15 genomes labeled with a *Brevundimonas* species name should be assigned to another *Brevundimonas* species (the above-mentioned strain TAR-002) or a proposed *Brevundimonas* taxon (the remaining 14), (ii) 7 genomes labeled as *Brevundimonas* sp. should be assigned to a known *Brevundimonas* species, and (iii) the remaining 25 genomes labeled as *Brevundimonas* sp. could be classified into 20 proposed taxa.

**TABLE 5:** Reference strains and closest species of the *Brevundimonas* taxa identified based on genome sequences in this study

Taxon	Reference strain	GenBank accession no.	Closest species	ANI (%)	isDDH value (%)
1	DSM 14878	GCA_014196125.1	*B. mediterranea*	94.79	57.30
2	DSM 23858	GCA_000421705.1	*B. naejangsanensis*	94.22	54.30
3	090558	GCA_014218725.1	*B. huaxiensis*	95.33	63.90
4	NBRC 12165	GCA_001592205.1	*B. vesicularis*	95.00	59.90
5	NBRC 12165	GCA_001592205.1	*B. vesicularis*	95.18	60.20
6	NCTC8545	GCA_900445995.1	*B. diminuta*	81.94	21.20
7	DSM 23960	GCA_014196335.1	*B. lenta*	85.92	25.60
8	DSM 4736	GCA_011927945.1	*B. alba*	84.90	24.60
9	NBRC 12165	GCA_001592205.1	*B. vesicularis*	95.29	62.40
10	DSM 23858	GCA_000421705.1	*B. naejangsanensis*	93.45	49.90
11	NBRC 12165	GCA_001592205.1	*B. vesicularis*	95.28	61.90
12	NCTC8545	GCA_900445995.1	*B. diminuta*	92.94	47.70
13	ATCC 15264	GCA_000144605.1	*B. subvibrioides*	86.54	28.80
14	090558	GCA_014218725.1	*B. huaxiensis*	88.06	30.80
15	HAMBI_262	GCA_003350205.1	*B. bullata*	89.96	36.20
16	NBRC 12165	GCA_001592205.1	*B. vesicularis*	94.38	55.80
17	NCTC8545	GCA_900445995.1	*B. diminuta*	93.52	47.90
18	090558	GCA_014218725.1	*B. huaxiensis*	89.28	34.50
19	NCTC8545	GCA_900445995.1	*B. diminuta*	82.37	21.90
20	090558	GCA_014218725.1	*B. huaxiensis*	95.24	62.00
21	DSM 23960	GCA_014196335.1	*B. lenta*	84.19	24.20
22	090558	GCA_014218725.1	*B. huaxiensis*	89.12	34.00
23	NBRC 12165	GCA_001592205.1	*B. vesicularis*	95.24	61.50
24	NBRC 12165	GCA_001592205.1	*B. vesicularis*	95.16	61.50
25	DSM 17329	GCA_011761985.1	*B. terrae*	81.80	22.10
26	HAMBI_262	GCA_003350205.1	*B. bullata*	92.24	43.10
27	NBRC 12165	GCA_001592205.1	*B. vesicularis*	94.09	56.00
28	DSM 23858	GCA_000421705.1	*B. naejangsanensis*	92.13	44.00
29	ATCC 15264	GCA_000144605.1	*B. subvibrioides*	81.17	21.10

### Virulence factors of *Brevundimonas*.

As strain 090558^T^ was recovered from a human blood culture and *Brevundimonas* is known as an opportunistic pathogen (14), we attempted to identify potential virulence factors of 090558^T^ and other *Brevundimonas* species. However, there is a lack of studies of *Brevundimonas* pathogenicity. We therefore used the Virulence Factor Database (VFDB) to predict potential virulence factors for all of the 21 *Brevundimonas* species with genome sequences of type strains available, including *B. huaxiensis*. Four potential virulence factors in the VFDB were identified in *B. huaxiensis*, i.e., *acpXL* (encoding acyl carrier protein of lipopolysaccharide [LPS]), *bvrR* (encoding the transcriptional regulatory protein BvrR), *icl* (encoding an isocitrate lyase), and a gene named Rv0440 (encoding the chaperonin GroEL) (Data Set S3). *acpXL* is also present in all other *Brevundimonas* species, while *bvrR* is absent only from Brevundimonas diminuta, and *icl* is absent only from Brevundimonas lutea (Data Set S3). Rv0440 is present in seven other *Brevundimonas* species (Brevundimonas aveniformis, Brevundimonas basaltis, Brevundimonas denitrificans, Brevundimonas fluminis, Brevundimonas halotolerans, Brevundimonas lenta, and Brevundimonas viscosa) (Data Set S3). No additional virulence factors were identified in the two major *Brevundimonas* species causing human infections, B. diminuta and *B. vesicularis* (14).

## DISCUSSION

In this study, we report a novel *Brevundimonas* species, *B. huaxiensis*, based on both phenotypic and genomic analyses. We identified that the genome of B. denitrificans strain TAR-002^T^ actually belonged to another strain of B. abyssalis. We then applied the updated taxonomic assignations to curate genome sequences deposited in GenBank with the label of *Brevundimonas* and found the presence of 29 taxa, which were different from the 21 known *Brevundimonas* species with available genome sequences of type strains. We also found that the species identification of more than half of the *Brevundimonas* genome sequences in GenBank needs to be corrected. The above-described findings suggest the complicated taxonomy of *Brevundimonas* and the need for careful curation of genomes labeled as *Brevundimonas*.

For the 29 taxa identified among *Brevundimonas* genomes in GenBank in this study, it is possible that some are actually one of the 12 known *Brevundimonas* species but with no genome sequences of type strains being available. However, most of the 29 taxa (at least 17 taxa) appear to actually represent novel *Brevundimonas* species, which are unnamed as they have not been characterized by phenotype methods and therefore warrant further studies.

Only half of all *Brevundimonas* genomes in GenBank have information on the isolation source available (see Data Set S1 in the supplemental material). Among the 7 *Brevundimonas* genomes that are labeled with humans as the source, 3 belonged to *B. diminuta*, and 1 each belonged to taxon 2 closest to Brevundimonas naejangsanensis ([Table 5]), taxon 17 (closest to *B. diminuta* [Table 5]), *B. vesicularis*, and *B. huaxiensis* (090558^T^). *B. diminuta* and *B. vesicularis* are known as the two major *Brevundimonas* species causing human infections (14). However, species identification in clinical microbiology laboratories is commonly based on phenotypic features, but it is well known that such phenotype-based approaches can cause misidentification and are unreliable for precise species identification(e.g., see references 20–22). Therefore, *B. diminuta* and *B. vesicularis* reported in clinical cases may actually belong to other *Brevundimonas* species. Even the genome label in the NCBI database may not be correct or needs to be updated. For instance, two genomes of *Brevundimonas* strains recovered from humans labeled as *B. diminuta* in GenBank actually belong to taxa 2 and 17, respectively (Data Set S1). Nonetheless, it appears that among the 34 *Brevundimonas* species, few are associated with human infections, including *B. diminuta* (23), *B. vesicularis* (24), B. vancanneytii (25), and *B. huaxiensis* (this study). The pathogenicities of these *Brevundimonas* species have not been characterized (14), and the prediction of virulence factors using the VFDB for *Brevundimonas* species is preliminary. The virulence of *Brevundimonas* warrants further studies.

We are aware of the limitations of this study. First, the phenotypic features and the fatty acid contents of strain 090558^T^ were compared with those of other *Brevundimonas* species reported in references rather than, ideally, in the same experiments. Second, as mentioned above, there are no genome sequences available for type strains of 12 known *Brevundimonas* species, although they are not closely related to strain 090558^T^. Third, there is only a single strain for the novel species identified here. Despite these limitations, the novel species status of *B. huaxiensis* can be established by the above-mentioned detailed analyses.

In conclusion, we identified and characterized a novel *Brevundimonas* species, *B. huaxiensis*, which is associated with human infection and is therefore of clinical relevance. *B. huaxiensis* can be differentiated from other *Brevundimonas* species by its ability to assimilate sodium acetate but not to utilize glucose, trypsin, or β-glucosidase. The genus *Brevundimonas* has 34 known species and at least 17 potential novel, unnamed species. Currently, few *Brevundimonas* species have been found to be associated with human infections. Genome sequencing of type strains of all *Brevundimonas* species is required to further untangle the taxonomic complexity of the genus. The proposed *Brevundimonas* taxa warrant further characterization using both phenotype- and genome-based approaches to establish their proper species assignations. More studies of the human-associated *Brevundimonas* species are required to understand their pathogenicity and epidemiology in clinical infections.

## MATERIALS AND METHODS

### Strain.

Strain 090558^T^ was recovered from the blood of a 46-year-old patient at West China Hospital of Sichuan University, Chengdu, China, in November 2019. Preliminary species identification was performed using Vitek II (bioMérieux, Marcy l’Etoile, France).

### Analysis of the 16S rRNA gene sequence.

Genomic DNA of strain 090558^T^ was prepared using a bacterial DNA kit (Tiangen, Beijing, China). The 16S rRNA gene was amplified by PCR using the universal primers 27F and 1492R (26) and Sanger sequencing for preliminary species identification. 16S rRNA gene sequences of related species were retrieved from GenBank and aligned with that of strain 090558^T^ using Clustal Omega (27). A maximum likelihood tree was inferred in RAxML v8.2.12 (28), and two more trees were inferred based on the neighbor-joining and maximum parsimony algorithms using MEGA v10.2.6 (29) with a 1,000-bootstrap test.

### Whole-genome sequencing and analysis.

Whole-genome sequencing of strain 090558^T^ was performed on the Illumina HiSeq X10 platform (Illumina, San Diego, CA, USA). JCM 11415^T^, the type strain of *B. nasdae*, had >99% 16S rRNA sequence identity with strain 090558^T^ but had no genome sequences or sequences of housekeeping genes for comparison. We therefore obtained *B. nasdae* strain JCM 11415^T^ from the Japan Collection of Microorganisms (JCM) via Shanghai Yansheng Ltd. and also sequenced the strain using the HiSeq X10 platform. Reads were *de novo* assembled into contigs using SPAdes v3.13.0 (30), applying the careful and auto-cutoff modes. The draft genome of strain 090558^T^ was compared with those of type strains of *Brevundimonas* species using the average nucleotide identity (ANI) based on BLAST analysis and *in silico* DNA-DNA hybridization (isDDH). ANI and isDDH values were calculated using JSpecies (16) and Genome-to-Genome Distance Calculator (formula 2) (17) with the recommended parameters and/or default settings, respectively. A ≥70.0% isDDH value (16, 17) and a ≥95 to 96% ANI value (16) were used as the cutoffs to define a bacterial species.

Whole-genome sequences of type strains of all *Brevundimonas* species (Table 2) were retrieved from GenBank. These genome sequences were annotated using Prokka v1.12 (31). Orthologues of these strains were identified using PIRATE v1.0.3 (https://github.com/SionBayliss/PIRATE) to represent the core genome. The gene sequences of the core genome were aligned and concatenated using MAFFT v7.313 (32) and AMAS v0.98 (33). A phylogenomic tree based on core genome sequences was then inferred using RAxML v8.2.12 (28) with the general-time-reversible (GTR) model plus gamma distribution and a 1,000-bootstrap test.

### Phenotypic characterization and *in vitro* susceptibility testing.

Growth on nutrient agar, tryptic soy agar, R2A agar, MacConkey agar, and blood heart infusion (BHI) agar (all from Hopebio, Qingdao, China) was examined at 35°C for 2 days. Cell motility was examined by observing bacterial growth and diffusion on a deep semisolid nutrient agar medium of 0.3% (wt/vol) agar (Hopebio). Anaerobic growth was examined by streaking the bacterial cultures on brain heart infusion agar plates and placing them in a GasPak EZ anaerobic bag (BD, Franklin Lakes, NJ, USA) at 30°C for 5 days. After incubation in R2A broth at 30°C for 3 days, flagella of strain 090558^T^ were observed with an H-7650 transmission electron microscope (Hitachi, Tokyo, Japan). The growth of strain 090558^T^ was examined in 5-ml aliquots of R2A broth dispensed into tubes (16-mm inner diameter) at temperatures of 4°C, 8°C, 18°C, 28°C, 32°C, 37°C, 42°C, 45°C, 48°C, and 50°C. Salt and pH tolerances were measured using R2A broth at 30°C for 5 days at different NaCl concentrations (0.5, 1, 2, 3, 4, 5, 7.5, 10, and 15% [wt/vol]) and various pHs (pH 4.0 to 12.0, in increments of 1.0 unit), respectively. A catalase activity test was conducted by examining the production of bubbles after the addition of a 3% (vol/vol) hydrogen peroxide solution, while oxidase activity was tested by using a 1% tetramethyl-*p*-phenylenediamine dihydrochloride solution. DNase activity was detected with 1 M HCl using DNase agar (Solarbio, Beijing, China) after 3 days of incubation at 30°C. Malonate, phenylalanine deaminase, and potassium cyanide (KCN) experiments were performed using biochemical identification tubes (Huankai, Guangzhou, Guangdong, China). Commercially available API 20NE and API Zym kits (bioMérieux) were used for testing biochemical features and enzyme activities.

MICs of amikacin, ampicillin, ampicillin-sulbactam, aztreonam, ceftriaxone, ceftazidime, cefepime, cefotaxime, cefuroxime, chloramphenicol, ciprofloxacin, colistin, imipenem, meropenem, piperacillin-tazobactam, sulfamethoxazole-trimethoprim, and tigecycline were determined by Vitek II using the broth microdilution method. Breakpoints defined by the CLSI (34) were applied, except for tigecycline, for which breakpoints defined by the European Committee on Antimicrobial Susceptibility Testing (EUCAST) (http://www.eucast.org/) were used.

### Fatty acid analysis.

The analysis of cellular fatty acids, quinones, and polar lipids was performed by the Guangdong Institute of Microbiology (Guangzhou, Guangdong). Briefly, fatty acid methyl esters were extracted and analyzed by gas chromatography according to the instructions of the Sherlock microbial identification system (MIDI Inc., Newark, DE, USA) as described previously (35, 36). Peaks were automatically integrated, and fatty acid proportions were calculated using the MIDI identification database RTSBA6 (version 6.00; MIDI Inc.).

### Virulence factor prediction.

Type strains of the genus *Brevundimonas* with genomes available (*n* = 20) as well as *B. huaxiensis* 090558^T^ and *B. nasdae* JCM 11415^T^ sequenced in this study were screened for the presence of virulence factors against 32,838 sequences (accessed on 6 March 2021) retrieved from data set B in the Virulence Factor Database (VFDB) (37) by a nucleotide similarity search using local BLAST (38). Hits with either coverage or identity of below 70% were discarded in the results.

### Curation of species identification for *Brevundimonas* genomes in GenBank.

We used txid41275 [Organism:exp] AND “latest” [filter] to search NCBI GenBank, and a total of 175 assemblies were available (accessed on 25 January 2021) in the genus *Brevundimonas*. We discarded 93 genomes that were labeled as “excluded from RefSeq” by NCBI GenBank due to either (i) being derived from a metagenome, (ii) having a fragmented assembly, (iii) having a genome length too small, or (iv) having many frameshifted proteins. Therefore, 82 *Brevundimonas* genome sequences (type strains of 22 species and 60 non-type strains) were included (Data Set S1), all of which were retrieved and then subjected to precise species identification using ANI and isDDH analyses as described above. Strains that have both a <70% isDDH value and a <96% ANI value with any known *Brevundimonas* species are likely to belong to a novel species, which is temporarily assigned a taxon here as the establishment of a novel species requires phenotypic characterization in addition to genome analysis.

### Data availability.

The draft genome sequence and the nearly complete 16S rRNA gene of strain 090558^T^ have been deposited in the DDBJ/EMBL/GenBank database under accession no. JABBJE000000000 and MT444005, respectively. The draft genome sequence of JCM 11415^T^ has been deposited in the DDBJ/EMBL/GenBank database under accession no. JAEPWN000000000.
