# Supplementary material for: Genome-Based Taxonomy of Brevundimonas with Reporting Brevundimonas huaxiensis sp. nov
Source: Microbiol Spectr. 2021 Jul 7;9(1):10.1128/spectrum.00111-21. doi: 10.1128/spectrum.00111-21 (PMC8552745; doi:10.1128/spectrum.00111-21)

**Supplementary files**

**Table S1.** The fatty acid contents of *B. huaxiensis* and closely related strains of the genus *Brevundimonas*

**Fig. S1** A neighbor-joining phylogenetic tree based on 16S rRNA gene sequences of strain 090558^T^ and type strains of *Brevundimonas* species.

**Fig. S2.** A maximum parsimony phylogenetic tree based on 16S rRNA gene sequences of strain 090558^T^ and type strains of *Brevundimonas* species.

**Fig. S3.** Transmission electron micrograph of strain 090558^T^.

**Dataset S1.** *Brevundimonas* genomes available in GenBank (accessed by 25-01-2021) included in analysis (in a separate file).

**Dataset S2.** ANI and isDDH values of *Brevundimonas* genomes available in GenBank (accessed by 25-01-2021) included in analysis (in a separate file).

**Dataset S3.** Virulence factors predicted using VFDB for type strains of *Brevundimonas* species with genome sequences available (in a separate file).

**Table S1.** The fatty acid contents of *B. huaxiensis* and closely related strains of the genus *Brevundimonas*

| **Fatty acid** | **1** | **2** | **3** | **4** | **5** | **6** |
| --- | --- | --- | --- | --- | --- | --- |
| Straight chain saturated |  |  |  |  |  |  |
| C_12:0_ 3-OH | 2.1 | 2.4 | 2.7 | 2.6 | 2.3 | 2.1 |
| C_14:0_ | 3.2 | 2.8 | 2.8 | 1.0 | 1.7 | TR |
| C_16:0_ | 25.2 | 15.9 | 21.2 | 16.8 | 17.9 | 16.2 |
| C_17:0_ | 1.2 | 6.2 | TR | 4.8 | 1.4 | 6.0 |
| C_18:0_ | 1.6 | TR | 1.3 | TR | 1.1 | TR |
| C_19:0_ cyclo *ω*8*c* | － | － | － | － | 9.6 | － |
| Unsaturated |  |  |  |  |  |  |
| C_17:1_*ω*8*c* | 1.8 | 3.0 | TR | 3.9 | TR | 9.5 |
| C_17:1_*ω*6*c* | 1.1 | 3.4 | TR | 3.2 | TR | 8.1 |
| 11-Methyl C_18:1_*ω*7*c* | 2.6 | 4.5 | 4.9 | 6.7 | TR | 4.4 |
| C_20:2_ *ω*6,9*c* | － | － | － | TR | TR | TR |
| Summed features* |  |  |  |  |  |  |
| 3 (C_16:1_*ω*7*c*)/(C_16:1_*ω*6*c*) | 4.0 | 4.4 | 5.9 | 9.1 | 3.3 | 13.1 |
| 8 (C_18:1_*ω*7*c*)/(C_18:1_*ω*6*c*) | 55.9 | 54.4 | 55.6 | 47.8 | 58.2 | 40.6 |

Strains: 1, *B. huaxiensis*; 2, *B. aurantiaca* KACC 12011^T^; 3, *B. vesicularis* KACC 11290^T^; 4, *B. intermedia* KACC 12012^T^; 5, *B. nasdae* KACC 12009^T^; 6, *B. mediterranea* KACC 12010T. Closely related species refers to those having >98.5% 16S rRNA gene sequence identity with *B. huaxiensis* 090558^T^ (Table 1).

Data for species other than *B. huaxiensis* are obtained from the reference[^2^](#_ENREF_2).

-, None; TR, trace amount (<1%). Note: minor fatty acids (<1 %) for *B. huaxiensis* are C_12:1_ 3-OH, iso-C_15:0_, iso-C_17:0_, iso-C_20:0_, summed feature1(C_13:0_ 3-OH/C_15:1_ iso-H), summed feature7(C_19:0_ cyclo *ω*10*c/*C_19:0_ cyclo19*ω*6). *Summed features represent fatty acids that could not be separated by the MIDI system.

**Fig. S1.** **A neighbor-joining phylogenetic tree based on 16S rRNA gene sequences of strain 090558^T^ and type strains of *Brevundimonas* species.** The tree was inferred using the maximum-likelihood method. Bootstrap values >70 % (based on 1,000 resamplings) are shown. Bar, 0.01 substitutions per nucleotide position.

**Fig. S2.** **A maximum parsimony phylogenetic tree based on 16S rRNA gene sequences of strain 090558^T^ and type strains of *Brevundimonas* species.** The tree was inferred using the maximum-likelihood method. Bootstrap values >70 % (based on 1,000 resamplings) are shown.

**Fig. S3.** Transmission electron micrograph of strain 090558^T^. Bar 1.0 $\mu$m.


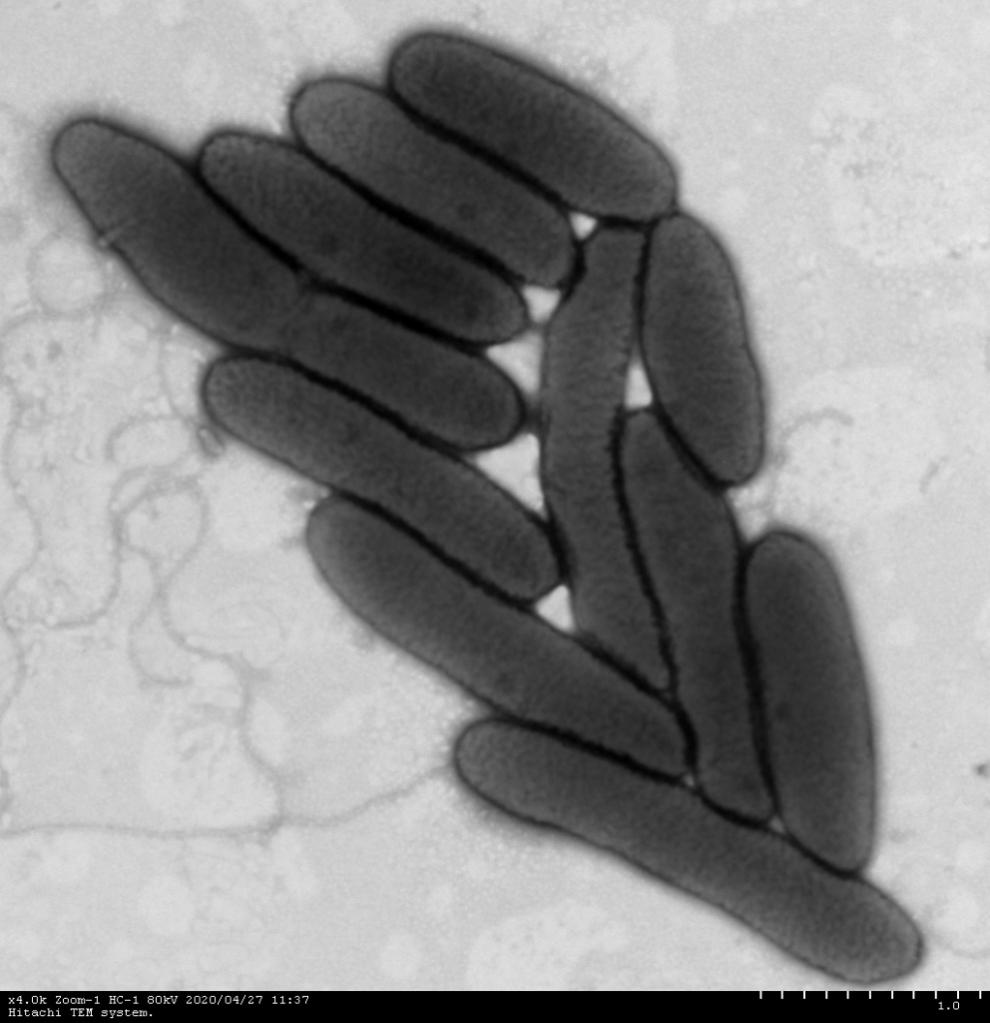

Supplement: Supplemental file 1 — Supplemental material. Download SPECTRUM00111-21_Supp_1_seq5.docx, DOCX file, 0.2 MB [file spectrum00111-21_supp_1_seq5.docx]
